# Elevated Preoperative Neutrophil-Lymphocyte Ratio Is Associated with Poor Prognosis in Hepatocellular Carcinoma Patients Treated with Liver Transplantation: A Meta-Analysis

**DOI:** 10.1155/2016/4743808

**Published:** 2015-12-30

**Authors:** Xiao-Dong Sun, Xiao-Ju Shi, Yu-Guo Chen, Chuan-Lei Wang, Qiang Ma, Guo-Yue Lv

**Affiliations:** Department of Hepatobiliary and Pancreatic Surgery, The First Hospital of Jilin University, Changchun, Jilin 130021, China

## Abstract

This study aims to investigate the prognostic value of neutrophil to lymphocyte ratio (NLR) in hepatocellular carcinoma (HCC) patients treated with liver transplantation (LT) through meta-analysis. Relevant articles were sought in PubMed, Embase, and Wangfang databases up to July 2015. A total of 1687 patients from 10 studies were included in this meta-analysis. Meta-analysis results showed that elevated NLR was significantly associated with poorer overall survival (OS) (HR = 2.71, 95% CI: 1.91–3.83) and poorer disease-free survival (DFS) (HR = 3.61, 95% CI: 2.23–5.84) in HCC patients treated with LT. Moreover, subgroup analysis showed the significant association between elevated preoperative NLR and poor prognosis was not altered by cutoff values of NLR or types of LT. Therefore, elevated preoperative NLR is associated with poor prognosis in HCC patients treated with LT. Preoperative NLR should be used to predict the prognosis of HCC after LT in our clinical work.

## 1. Introduction

Hepatocellular carcinoma (HCC), the most common primary malignancy of the liver, is the second common cause of cancer-related deaths worldwide, and its incidence is increasing steadily in America [1, 2]. According to GLOBOCAN 2012, an estimated 782,500 new liver cancer cases and 745,500 deaths occurred worldwide during 2012, with China alone accounting for about 50% of the total number of cases and deaths [3]. Liver transplantation (LT) presents as an attractive treatment modality for HCC, with the advantage of moving tumor totally, correcting underlying cirrhosis, and reducing the risk of postoperative liver failure [4]. However, the prognosis of transplant recipients remains unsatisfactory with 5-year survival rate of 84%, though advancements have been achieved in the managements of HCC patients treated with LT [5]. Meanwhile, there are very few preoperative markers that can be used to predict the prognosis of transplant recipients, except the prolonged waitlist time and high alpha-fetoprotein (AFP) level [6]. Therefore, it is essential to identify marker especially preoperative factors, which can be used to predict the prognosis of HCC patients after LT.

Nowadays, increased neutrophil to lymphocyte ratio (NLR) before initial treatments, which represents the systemic inflammatory response, has been proved to be associated with poor prognosis in diverse malignancies, such as gastrointestinal cancers (including esophageal cancer, gastric cancer, colorectal cancer, and pancreatic cancer), urological cancers, and lung cancer [7–13]. However, as a matter of contradictory results as well as the small sample size in solitary study, the current opinion of NLR as the prognostic marker in HCC patients treated with LT is still inconclusive.

Therefore, we conducted this meta-analysis from eligible studies to investigate the relationship between preoperative NLR and the prognosis of HCC patients. Meanwhile, we also conducted subgroup analysis to assess the prognostic role of NLR in HCC patients according to cutoff values of NLR and types of LT.

## 2. Materials and Methods

### 2.1. Literature Search Strategy

We searched the PubMed, Embase, and Wangfang databases for relevant articles up to July 2015. The search terms included (“neutrophil to lymphocyte ratio”, “neutrophil-lymphocyte ratio”, “NLR”, “neutrophil/lymphocyte ratio”), (“hepatocellular carcinoma”, “HCC”), and (“liver transplantation”). The search strategy used in PubMed is as follows: “(Liver transplantation [Title/Abstract]) AND ((((Neutrophil-lymphocyte ratio) OR Neutrophil lymphocyte ratio) OR Neutrophil/lymphocyte ratio) OR Neutrophil : lymphocyte ratio)”. Furthermore, we also scanned reference lists of retrieved studies and reviews for additional available studies.

### 2.2. Selection and Exclusion Criteria

Studies included in the meta-analysis had to meet the following criteria: (1) HCC was diagnosed by pathological methods, (2) NLR was tested before LT, (3) the correlation of NLR with overall survival (OS) and/or disease-free survival (DFS) was investigated, and (4) the values of hazard ratio (HR) with corresponding 95% confidence interval (CI) were provided directly or could be calculated indirectly. The following studies were excluded from the analysis: (1) letters, reviews, comments, and conference articles, (2) studies with NLR tested after LT, and (3) articles without deficit cutoff value of NLR. Regarding multiple publications from the same population, only the most recent or the most complete study was included in the analysis.

### 2.3. Data Extraction

Two investigators (Sun XD, Shi XJ) extracted the main characteristics from each included study independently, including first author, origin of population, year of publication, study sample size, age (mean/median), type of liver transplantation (e.g., living donor liver transplantation, deceased donor liver transplantation), tumor stage (under/over Milan criteria), immunosuppressive agents, cutoff values of NLR, study endpoints (OS, DFS, and survival rate), HR with corresponding CI, HR source (direct, available data, or Kaplan-Meier curve), and follow-up time. If both univariate and multivariate analysis results were reported, we used the latter one. If HRs were not provided directly in the article, the total numbers of observed deaths/cancer recurrences and the numbers of samples in each group were extracted to calculate HRs [14]. Besides, we also used Engauge Digitizer version 4.1 (http://sourceforge.net/) to read the Kaplan-Meier curves when the data above were not available either; then, we calculated the HRs with their corresponding CIs as before [14]. After this process, extracted data were then cross-checked between the two authors to rule out any discrepancy. In case, disagreements were discussed by the authors and resolved by consensus.

### 2.4. Quality Assessment of Primary Studies

In this meta-analysis, Newcastle-Ottawa Scale (NOS) criteria were used to assess the methodological quality of included studies [15]. The NOS criteria are scored based on three aspects: (1) subject selection, (2) comparability of subject, and (3) clinical outcome. NOS scores range from 0 to 9, and a score ≥6 indicates a high quality. Two investigators (Chen Y. G. and Wang C. L.) independently assessed the quality of the 10 included studies, and the discrepancies were solved by consensus.

### 2.5. Data Analysis

The statistical analysis was carried out using STATA version 10.0 and SPSS version 13.0. On the one hand, pooled HR with 95% CI was used to estimate the effect of elevated NLR on OS and DFS in HCC patients after LT. A combined HR > 1 indicated a poor outcome for patients with elevated NLR, while HR < 1 indicated a favorable outcome for elevated NLR. On the other hand, pooled odds ratio (OR) with 95% CI was used to assess the impact of elevated NLR on survival rate of HCC patients after LT. A combined OR > 1 indicated a favorable outcome, while a combined OR < 1 indicated a poor outcome. The prognostic value of NLR in HCC after LT was regarded as significant when the combined 95% CI did not overlap 1 unit. Heterogeneity among the studies was determined by Chi-square test and *Q* test [16]. If heterogeneity was significant (*P* < 0.1 or *I* ^2^ > 50%), random-effect model was used. Otherwise, fixed-effect model was used. All *P* values were two-tailed with a significant level at 0.05.

## 3. Results

### 3.1. Literature Information

**Figure 1:**
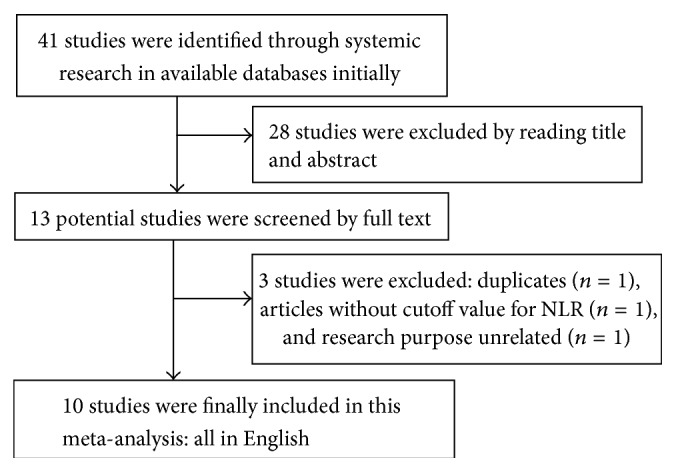
Flowchart of searching relevant studies used in this meta-analysis.

Initially, 41 articles were identified according to the search strategies as described before. Then, 28 articles above were excluded by reading title and abstract. Of the 13 ones that remained, 3 articles were excluded by full text: duplicate (*n* = 1), article without cutoff value of NLR (*n* = 1), and unrelated research purpose (*n* = 1). Consequently, 10 articles were included in this final meta-analysis [17–26].  Figure 1 shows a flow diagram of the selection process for relative articles.

### 3.2. Study Characteristics

The baseline characteristics of all included studies were summarized in Table 1. The 10 retrospective studies were carried out in 5 countries (1 in Korea, 4 in Japan, 2 in China, 2 in America, and 1 in Italy), and they were published between 2009 and 2014. A total of 1687 patients were included, and the median number of all included studies was 159 (ranging from 101 to 280). The cutoff values for elevated NLR ranged from 3 to 6, among which 2 studies set this value as 3.0, 4 studies set this value as 4.0, 3 studies set this value as 5.0, and only 1 study set this value as 6.0. Regarding transplantation types, living donor liver transplantation (LDLT) was used in 6 studies, deceased donor living transplantation (DDLT) was used in 3 studies, and both of these types were used in 1 study. According to the NOS criteria, the mean score of the included studies was 8 (ranging from 6 to 9), which indicated high methodological quality (Table 2).

### 3.3. Meta-Analysis for OS

**Figure 2:**
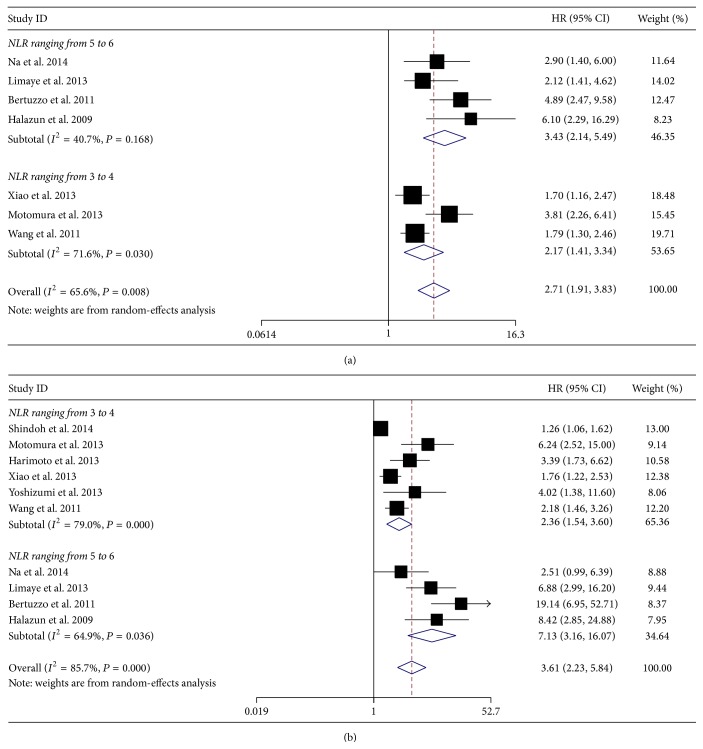
Meta-analysis for the correlation of neutrophil to lymphocyte ratio (NLR) with overall survival (OS) (a) and disease-free survival (DFS) (b) in hepatocellular carcinoma (HCC) patients treated with liver transplantation (LT).

Totally, there are seven studies with 1292 HCC patients investigating the association between preoperative NLR and OS after LT. Since heterogeneity was found among these studies (*I* ^2^ = 65.6%, *P* = 0.008), random-effect model was used to calculate the combined HR. According to this model, elevated preoperative NLR was significantly associated with poor OS (HR = 2.71, 95% CI: 1.91–3.83, and *P* = 0.000), suggesting that elevated preoperative NLR was an indicator of poor survival rate in HCC patients treated with LT (Figure 2(a)).

### 3.4. Meta-Analysis for DFS

Meanwhile, there are ten studies with 1687 HCC patients investigating the prognostic value of preoperative NLR on DFS of HCC patients after LT. There was heterogeneity between these studies, so random-effect model was used to calculate the combined HR (HR = 3.61, 95% CI: 2.23–5.84, and *P* = 0.000) (Figure 2(b)). These results above demonstrated that elevated preoperative NLR was significantly associated with poor DFS, indicating elevated preoperative NLR was an indicator of early tumor recurrence rate in HCC patients treated with LT.

### 3.5. Meta-Analysis for Survival Rate

**Figure 3:**
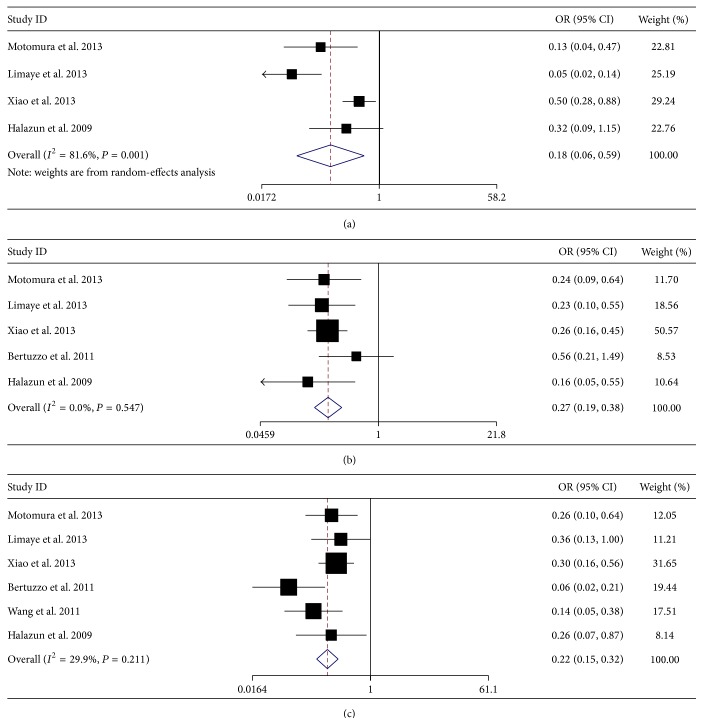
Meta-analysis for the correlation of neutrophil to lymph node ratio (NLR) with 1-year overall survival (OS) rate (a), 3-year OS rate (b), and 5-year OS rate (c) in hepatocellular carcinoma (HCC) patients treated with liver transplantation (LT).

**Figure 4:**
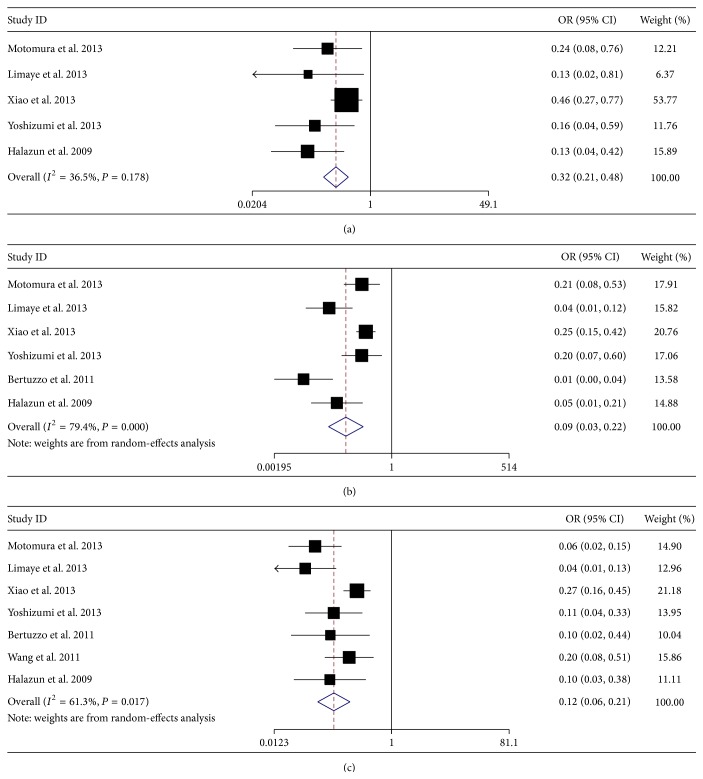
Meta-analysis for the correlation of neutrophil to lymphocyte ratio (NLR) with 1-year disease-free survival (DFS) rate (a), 3-year DFS rate (b), and 5-year DFS rate (c) in hepatocellular carcinoma (HCC) patients treated with liver transplantation (LT).

In addition, we also investigated the prognostic value of preoperative NLR on different survival rate. In detail, four studies with 748 patients reported 1-year OS rate, five studies with 928 patients reported 3-year OS rate, six studies with 1019 patients reported 5-year OS rate, five studies with 852 patients reported 1-year DFS rate, six studies with 1022 patients reported 3-year DFS rate, and seven studies with 1219 patients reported 5-year DFS rate. Meta-analysis results showed that elevated NLR was significantly associated with poor 1-year OS rate (OR = 0.18, 95% CI: 0.06–0.59, and *P* = 0.004, random-effect model) (Figure 3(a)), poor 3-year OS rate (OR = 0.27, 95% CI: 0.19–0.38, and *P* = 0.000, fixed-effect model) (Figure 3(b)), poor 5-year OS rate (OR = 0.22, 95% CI: 0.15–0.32, and *P* = 0.000, fixed-effect model) (Figure 3(c)), poor 1-year DFS rate (OR = 0.32, 95% CI: 0.21–0.48, and *P* = 0.000, fixed-effect model) (Figure 4(a)), poor 3-year DFS rate (OR = 0.09, 95% CI: 0.03–0.22, and *P* = 0.000, random-effect model) (Figure 4(b)), and poor 5-year DFS rate (OR = 0.12, 95% CI: 0.06–0.21, and *P* = 0.000, random-effect model) (Figure 4(c)).

### 3.6. Subgroup Analysis for OS and DFS

When the cutoff value of NLR was set ranging from 3.0 to 4.0, elevated NLR was significantly associated with poor OS (HR = 2.17, 95% CI: 1.41–3.34, and *P* = 0.000) and poor DFS (HR = 2.36, 95% CI: 1.54–3.60, and *P* = 0.000). Then, when the cutoff value of NLR was set higher (ranging from 5.0 to 6.0), elevated NLR was significantly associated with poorer OS (HR = 3.43, 95% CI: 2.14–5.49, and *P* = 0.000) and poorer DFS (HR = 7.13, 95% CI: 3.16–16.07, and *P* = 0.000). On the contrary, the significant association between elevated NLR and prognosis in HCC patients was not altered by LT types (Table 3). Therefore, elevated preoperative NLR was associated with poor prognosis in HCC patients treated with LT, despite the cutoff values of NLR and types of LT.

## 4. Discussion

The relationship between inflammation and cancer was hypothesized by Rudolph Virchow back in 1850s [27]. Consistently, epidemiologic studies estimate that over 20% of all human cancer cases are associated with chronic inflammation [28]. This association has been observed in various types of gastrointestinal malignancies, including persistent reflux esophagitis with esophageal cancer,* Helicobacter pylori* infection with gastric cancer, inflammatory bowel disease with colorectal cancer, hepatitis B/C virus infection with liver cancer, and chronic pancreatitis with pancreatic cancer [29]. It is revealed that inflammation-induced carcinogenesis is caused by several processes, such as genotoxicity, aberrant tissue repair, proliferative responses, invasion, and metastasis, through influencing the level of the transcription factors signal transducer and activator of transcription 3 (STAT3) and nuclear factor-*κ*B (NF-*κ*B) [30]. Besides, systemic inflammation is associated with increased weight loss and poorer performance status, which may be an important etiological factor in the nutritional and functional decline of the advanced cancer patient [31].

Recently, hematological markers of systemic inflammation, such as NLR, C-reactive protein (CRP), and platelet to lymphocyte ratio (PLR), have been shown to have prognostic value in cancer patients [7]. However, the prognostic value of NLR in HCC patients treated with LT remains inconclusive. Coincidentally, there are few preoperative markers that can be used to predict the prognosis of HCC patients treated with LT, except the prolonged waitlist time and high AFP [6]. Therefore, there is great interest in evaluating the prognostic role of preoperative NLR in HCC patients treated with LT, especially using these hematological markers of systemic inflammation as described above.

Previously, a meta-analysis showed that elevated NLR was significantly associated with poor prognosis for patients with HCC. However, only six studies about LT were included in this meta-analysis, which was analyzed as subgroup [32]. Therefore, our study should be the first meta-analysis to assess the prognostic value of NLR in HCC patients treated with LT as a whole. In our meta-analysis, we included 10 studies with a total of 1687 patients, and we investigated the prognostic value of NLR in HCC patients by OS, DFS, and different OS/DFS rates. Meta-analysis results demonstrated that elevated preoperative NLR was significantly associated with poorer OS (HR = 2.71, 95% CI: 1.91–3.83), poorer DFS (HR = 3.61, 95% CI: 2.23–5.84), and decreased 1/3/5 OS/DFS rate. Subgroup analysis showed that there was a positive correlation between the increase in cutoff value of NLR and the increase of HR for prognosis, and the significant correlation between NLR and prognosis was not altered by LT types. These results above suggested that elevated preoperative NLR can be used as an indicator of poor survival rate and early tumor recurrence rate in HCC patients treated with LT.

However, there are several limitations in this current meta-analysis that should be acknowledged. The first and foremost thing is heterogeneity, which was found in the main meta-analysis with OS and DFS. The cutoff values for elevated NLR, LT types, tumor stages, immunosuppressive therapeutic methods, and follow-up time are so diverse that these factors may account for the heterogeneity. Secondly, publication bias was observed in the meta-analysis with OS and DFS (not shown in this paper). One reason may be that some articles in another language or from other databases were not achieved; the other reason may be that some articles without explicit cutoff value of NLR were excluded in the analysis. Last but not least, it is not available to conduct subgroup analysis according to the potential causes of HCC (HCC virus or metabolic disease) though we want to exclude the confounding factor of hepatitis virus, which is because the potential causes for HCC in each included study were diverse or not explicit. Based on these limitations above, the pooled HRs/ORs calculated in our meta-analysis may be just estimation, and our results should be substantiated by more additionally prospective and large-scale studies.

## 5. Conclusion

Elevated preoperative NLR is associated with poor prognosis in HCC patients treated with LT, and preoperative NLR should be used as a marker to predict the survival rate and tumor recurrence rate in HCC patients after LT in our clinical work.

## Figures and Tables

**Table 1:** Characteristics of included studies in this meta-analysis.

First author [ref.]	Country	Year	Number (M/F)	Age (years)	LT types	MC (U/O)	Immunosuppressive agents	Study design	Cutoff for LNR (> versus <)	Study endpoints	HR with 95% CI (analysis methods)	Sources	Follow-up time (months)
Na [17]	Korea	2014	224 (184/40)	Mean 51.9 ± 6.9	LDLT	141/83	TAC/CyA + MMF + prednisolone	R	6.0 (27/197)	OS, DFS	OS (M), 2.90 (1.40–6.00)	Direct	Median 68 (6–139)
											DFS (M), 2.51 (0.99–6.39)	Direct	

Shindoh [18]	Japan	2014	124 (98/26)	Median 56 (37–67)	LDLT	80/44	TAC/CyA + steroid with/without MMF	R	3.0 (61/62)	RFS	RFS (M), 1.26 (1.06–1.62)	Direct	Median 101.9 (3.5–165.4)

Xiao [19]	China	2013	280 (249/31)	Mean 46.5 (20.5–69.1)	Mixed	NA	TAC/CyA + steroids with/without azathioprine and MMF	R	4.0 (114/166)	OS, RFS, and 1/3/5 years OS/RFS	OS (M), 1.70 (1.16–2.47)	Direct	Mean 31.56 (13.2–144.0)
											DFS (M), 1.76 (1.22–2.53)	Direct	

Limaye [20]	America	2013	160 (130/30)	Mean 55.4	DDLT	144/16	TAC + steroid with/without MMF	R	5.0 (28/132)	OS, DFS, and 1/3/5 years OS/RFS	OS (U), 2.12 (1.41–4.62)	Direct	Mean 38 (1–116)
											RFS (U), 6.88 (2.99–16.20)	Direct	

Motomura [21]	Japan	2013	158 (92/66)	Mean 57	LDLT	94/64	TAC/CyA + MMF	R	4.0 (26/132)	OS, RFS, and 1/3/5 years OS/RFS	OS (U), 3.81 (2.26–6.41)	Curve	Median 40.3
											RFS (M), 6.24 (2.52–15.0)	Direct	

Harimoto [22]	Japan	2013	167 (NA/NA)	NA	LDLT	105/62	TAC/CyA + steroid and/or MMF	R	4.0 (26/141)	RFS	RFS (U), 3.39 (1.73–6.62)	Data	Median 46.8

Yoshizumi [23]	Japan	2013	104 (63/41)	Median 58.0 (41–72)	LDLT	52/52	TAC/CyA + steroid and/or MMF	R	4.0 (21/83)	RFS, 1/3/5 years RFS	RFS (M), 4.02 (1.38–11.6)	Direct	Median 57.9

Wang [24]	China	2011	101 (92/9)	Mean 48.4 (27–72)	DDLT	36/65	Calcineurin inhibitors and steroids	R	3.0 (33/68)	OS, DFS, and 5 years OS/RFS	OS (U), 1.79 (1.30–2.46)	Curve	Mean 34.2 (4.56–74.64)
											DFS (U), 2.18 (1.46–3.26)	Curve	

Bertuzzo [25]	Italy	2011	219 (186/33)	Median 57 (12–69)	DDLT	138/81	TAC/CyA (majority)	R	5.0 (23/147)	OS, RFS, and 3/5 years OS/RFS	OS (M), 4.89 (2.47–9.58)	Direct	Median 40 (1–146)
											RFS (M), 19.14 (6.95–52.71)	Direct	

Halazun [26]	America	2009	150 (119/31)	Mean 57.1 (29–74)	LDLT	104/46	TAC/CyA + steroid + MMF	R	5.0 (13/137)	OS, DFS, and 1/3/5 years OS/DFS	OS (M), 6.10 (2.29–16.29)	Direct	Mean 37.2 (13.2–82.8)
											DFS (M), 8.42 (2.85–24.88)	Direct	

M/F, male : female; NA, not available; LT, liver transplantation; LDLT, living donor liver transplant; DDLT, deceased donor liver transplant; MC, Milan criteria; R, retrospective; TAC, tacrolimus; CyA, cyclosporine; MMF, mycophenolate mofetil; NLR, neutrophil to lymphocyte ratio; OS, overall survival; DFS, disease-free survival; RFS, recurrence-free survival; U, univariate analysis; M, multivariate analysis; and curve, Kaplan-Meier curve.

**Table 2:** Newcastle-Ottawa quality for included studies in this meta-analysis.

First author and year [ref.]	Selection (score)				Comparability (score)		Outcome (score)			Total score
	Representativeness of exposed	Selection of nonexposed	Ascertainment of exposure	No interest before study	Study design (cohort study)	Control for other confounding factors	Assessment of outcome	Follow-up time long enough (>5 years)	Adequacy number of follow-ups (>80%)	
Na 2014 [17]	1	1	1	1	1	1	1	1	0	8
Shindoh 2014 [18]	1	1	1	0	1	1	1	1	1	8
Xiao 2013 [19]	1	1	1	0	1	1	1	1	1	8
Limaye 2013 [20]	1	1	1	1	1	0	0	1	1	7
Motomura 2013 [21]	1	1	1	0	1	0	1	1	1	7
Harimoto 2013 [22]	1	1	1	0	1	0	0	1	1	6
Yoshizumi 2013 [23]	1	1	1	1	1	1	1	1	1	9
Wang 2011 [24]	1	1	1	0	1	0	1	1	1	7
Bertuzzo 2011 [25]	1	1	1	0	1	1	0	1	1	7
Halazun 2009 [26]	1	1	1	0	1	1	1	1	1	8

**Table 3:** Subgroup analysis for the association between elevated preoperative NLR and prognosis of HCC patients treated with LT.

Study endpoints	Variables	Number of studies	Number of patients	HR (95% CI)	*P* value	Heterogeneity	
						*I* ^2^ (%)	*P* value
OS	Total	7	1292	2.71 (1.91–3.83)	0.000	65.6	0.008
	NLR range						
	3.0–4.0	3	539	2.17 (1.41–3.34)	0.000	71.6	0.030
	5.0–6.0	4	753	3.43 (2.14–5.49)	0.000	40.7	0.168
	LT types						
	LDLT	3	532	3.79 (2.57–5.60)	0.000	0.0	0.491
	DDLT	3	480	2.50 (1.43–4.37)	0.001	71.1	0.031
	Mixed	1	280	1.70 (1.17–2.48)	0.006	—	—

DFS	Total	10	1687	3.61 (2.23–5.84)	0.000	85.7	0.000
	NLR range						
	3.0–4.0	6	934	2.36 (1.54–3.60)	0.000	79.0	0.000
	5.0–6.0	4	753	7.13 (3.16–16.07)	0.000	64.9	0.036
	LT types						
	LDLT	6	927	3.38 (1.65–6.94)	0.001	84.0	0.000
	DDLT	3	480	6.19 (1.70–22.56)	0.006	89.2	0.000
	Mixed	1	280	1.76 (1.22–2.53)	0.002	—	—

NLR, neutrophil to lymphocyte ratio; HCC, hepatocellular carcinoma; LT, liver transplantation; OS, overallsurvival; DFS, disease-free survival; LDLT, living donor liver transplantation; DDLT, deceased donor living transplantation; HR, hazard ratio; and CI, confidence interval.
